# The new Lyon brace (ARTBrace). New concepts of scoliosis correction

**DOI:** 10.1186/1748-7161-9-S1-O56

**Published:** 2014-12-04

**Authors:** Jean Claude de Mauroy, Sophie Pourret

**Affiliations:** 1 Clinique du Parc, Lyon, France; 2 Lecante, Lyon, France

## Design

The ARTbrace is a new Asymmetric, Rigid polycarbonate, Torsion brace constructed with two asymmetrical lateral polycarbonate pieces connected posteriorly at the midline by a vertical incurved bar. Both anterior and lower closures are rigid, the upper third is Velcro. The aim is to build this brace in a simple custom way.

## Method

The first concept is the mathematical model of circled helicoid. In the torso column, the generating circle is perpendicular to the axis.

To obtain a torso column in the opposite side of scoliosis, the superposition of three electronic instantaneous full 3D moldings are necessary:

- First molding in self active axial elongation for pelvis and shoulders

- Second molding in lumbar shift and physiological lordosis for the lumbar spine

- Third molding in thoracic shift and physiological kyphosis for the thoracic spine.

The 3 moldings are superposed with specific software OrtenShape.

The second concept is the wrench and bolt principle. No more pressure and expansion, no more push and pull, scoliosis is untwisted by the brace.

The third principle according to Panjabi is the coupled motion behavior of the spine. When the spine is in a flexed or extended position (non-neutral) sidebending to one side will be accompanied by rotation to the same side correcting scoliosis rotation. Molding is 2D but correction is 3D.

These new concepts can avoid the plaster cast or major modifications of the mold like the Chêneau brace.

## Results

Economics: no more plaster cast, no more hospitalization, the life of the brace is greater than that of the plaster.

Efficiency: the brace is adjustable in the frontal plane, an additional correction by internal pad is easy (like Sforzesco).

Aesthetics: the brace is transparent, almost invisible under clothing.

Hygiene: a daily 15-minute shower is possible.

Lightness: No more 5-7 kg plaster cast.

Originality: This is the first untwisting brace of the whole spine in three planes of space.

Simplicity: anyone can make a frontal bending with lordosis or kyphosis, no major correction of the positive mold, a single setting, the protocol is identical to that of plaster cast.

Tolerance: Polycarbonate is biologically well tolerated.

Universality: It is possible to correct Hyperkyphosis like hypokyphosis.

